# Effect of parents' on menstrual hygiene in rural India

**DOI:** 10.6026/973206300214311

**Published:** 2025-12-15

**Authors:** Amita Singh, Satyalakshmi Komarraju, Sathyanath Dasrathan, Shrikanth Muralidharan, Himel Mondel

**Affiliations:** 1 Department of Physiology, Uttar Pradesh University of Medical Sciences, Saifai, Lachwai, Uttar Pradesh, India; 2 Department of Naturopathy and Yoga, National Institute of Naturopathy, Ministry of Ayush, Government of India, Pune - 411001, India; 3 Department of Natural Therapeutics, Nisarggram, National Institute of Naturopathy, Ministry of Ayush, Government of India, Pune - 411001, India; 4 Department of Research, National Institute of Naturopathy, Ministry of Ayush, Government of India, Pune - 411001, India; 5 Department of Physiology, All India Institute of Medical Sciences, Deoghar, Jharkhand - 814152, India

**Keywords:** Adolescent, hygiene, menstrual hygiene products, sanitation, menstruation, schools, educational status

## Abstract

Parent's education on menstrual hygiene affects the practices of girls especially in the rural areas. Therefore, it is of interest to explore the relationship between parental and menstrual hygiene practices in rural areas of Etawah, Uttar Pradesh, India. Hence, a cross-sectional study was conducted among 165 school-going girls aged 10-19 years in rural Etawah. No statistically significant association was found between parental education and access to menstrual hygiene products or challenges faced. Inadequate hygiene facility was high among all groups irrespective of parent's education. Thus, we show that parental education alone does not significantly impact menstrual hygiene practices in this rural setting.

## Background:

Menstrual hygiene is a critical component of adolescent health, yet it remains a neglected issue in many rural regions of India due to cultural taboos, inadequate infrastructure and limited awareness [1]. The ability to manage menstruation with dignity and safety depends not only on access to products and sanitation but also on social support and education [2]. Among the various influencing factors, parental education, especially that of mothers, plays a significant role in shaping a girl's knowledge, attitudes and practices regarding menstruation [3]. In communities where parents lack formal education, discussions about menstrual health are often absent and misconceptions are likely to persist, further contributing to poor hygiene practices [4]. Higher education contributes to improved menstrual hygiene practices, including greater access to sanitary products, increased likelihood of using disposable pads and better understanding of menstrual physiology [5]. Studies have shown that girls in rural settings often lack adequate information before menarche, face taboos around discussing menstruation and encounter inconsistent access to products and privacy for menstrual management [5, 6]. These broader determinants form the context within which parental education may or may not exert influence and highlight why examining its specific role is important. With this background, this study aims to explore how parents' educational status impacts menstrual hygiene among adolescent girls in rural areas of Etawah district, Uttar Pradesh. Understanding these dynamics can inform targeted interventions and policy measures that empower both parents and daughters, ensuring healthier and more equitable outcomes in rural settings. Therefore, it is of interest to report the influence of parental knowledge, especially mothers on the menstrual hygiene of girls in a rural area of India.

## Methods:

This cross-sectional, questionnaire-based study was conducted among school-going girls aged 10 - 19 years in rural areas of Etawah District, Uttar Pradesh, India. The sample size was determined using a standard formula based on an expected period poverty prevalence of 12.3%, a confidence level of 95% and a 5% margin of error and calculated sample size was 165 [5]. Inclusion criteria include female students within the specified age group who are willing to participate and provide informed consent (or assent with parental consent for minors). Data were collected using a structured questionnaire covering demographics, menstrual hygiene practices and period poverty experiences. Ethical approval was obtained from Uttar Pradesh, University of Medical Sciences. Data analysis involved descriptive statistics, chi-square tests and regression models to explore the association between period poverty and mental health. For analysis, R Studio 2024.12.1 (Posit Software, PBC, Boston, MA).

## Results:

A total of 165 girls participated in the study. The parents of the girls had various level of education with 21 had no formal education, 79 had primary education, 45 had secondary education and 20 had college/university education. Figure 1 (see PDF) shows education level does not have a significant effect (P = 0.15) on accessibility, meaning the two variables appear to be independent of each other. The difficulties according to education level are showing in Figure 2 (see PDF). The regression suggests negative trend higher education levels are associated with fewer reported challenges. However, the association is not statistically significant (P = 0.225).

## Discussion:

The study observed that the level of parental education did not show a statistically significant relationship with either access to menstrual hygiene products or the range of menstrual hygiene challenges faced by adolescent girls. Although a general trend suggested that girls from families with higher educational backgrounds reported fewer challenges, this trend did not reach statistical significance. A study found that higher education of mothers was associated with better menstrual hygiene; [7] however; we could not find any significant relation. The absence of a strong association may reflect the complex interplay of factors influencing menstrual hygiene. While parental education is expected to shape awareness and attitudes, it may be insufficient in isolation to ensure adequate access to hygiene products or overcome the social and infrastructural barriers present in rural communities [8]. The overall poor hygiene practice is seen all over the states in rural areas [9]. Poor sanitation infrastructure in all education groups was prevalent. Hence, the infrastructure is more influential than education, which was also reported by study by Madhu *et al.* [10]. In order to improve menstrual hygiene practices in rural settings requires a multi-pronged approach that goes beyond enhancing educational attainment. Interventions should address physical access to products, availability of girl-friendly sanitation infrastructure, sanitary pads and community-level education to challenge taboos [10, 11]. The study is limited by its scope and design. It was conducted in a specific rural region, which may limit the generalizability of its findings to other geographic or socio-economic contexts.

## Conclusion:

We show that menstrual hygiene challenges among adolescent girls in rural areas persist regardless of parental education levels. Enhancing access to menstrual hygiene products, improving sanitation facilities and addressing deep-rooted social taboos through community engagement and school-based programs are crucial steps toward ensuring dignified menstrual health for all girls.
